# The effect of backpack load on intersegmental motions of the foot and plantar pressure in individuals with mild flatfoot

**DOI:** 10.1186/s13047-022-00579-8

**Published:** 2022-10-15

**Authors:** Min Gyu Kyung, Ppu Ri Bak, Jong Wook Lim, Dong-Oh Lee, Gil Young Park, Dong Yeon Lee

**Affiliations:** 1 Department of Orthopedic Surgery, Seoul National University Hospital, 101 Daehak-ro, Jongno-gu, Seoul, 03080 Republic of Korea; 2 Seoul National University College of Medicine, Seoul, Republic of Korea; 3 Department of Orthopedic Surgery, SNU Seoul Hospital, Seoul, Republic of Korea; 4 Department of Orthopedic Surgery, Barunsesang Hospital, Seongnam, Gyeonggi-do Republic of Korea; 5 Department of Orthopedic Surgery, Seoul National University College of Medicine, 101 Daehak-ro, Jongno-gu, Seoul, 03080 Republic of Korea

**Keywords:** Backpack loading, Flat foot, Intersegmental motion, Multi-segment foot model, Pedobarography

## Abstract

**Background:**

The feet play an essential role in shock absorption, and foot posture is closely related to gait. The compensatory mechanism under heavy-load conditions in individuals with mild flatfoot is poorly understood. In the authors’ country, individuals with mild flatfoot are drafted as active-duty soldiers and participate in military rucking wearing heavy backpacks. This study investigated the effect of backpack load on gait and foot plantar pressure and possible differences in participants with mild flatfoot. The average weight of the backpack during military rucking (approximately 20 kg), was simulated in this study.

**Methods:**

This study prospectively enrolled 30 healthy young males, divided into a control group (CON, *n* = 15) and a mild low-arched group (MLA, *n* = 15), based on the presence of flatfoot. Segmental foot kinematics were evaluated using a three-dimensional multi-segment foot model, and gait data of the temporal and spatial parameters were obtained. The dynamic plantar pressure was simultaneously measured using a pedobarography platform with gait trials. The protocol was repeated with all participants wearing 20 kg backpacks. Comparisons between the baseline and loaded states, as well as comparison between groups, were conducted.

**Results:**

Although the cadence, gait speed, and stride length decreased in the loaded condition, step time and proportion of the stance phase increased in both groups. Although the MLA group showed more supinated and abducted positions of the forefoot and more pronated positions of the hindfoot than the CON group, the change in intersegmental foot and ankle motion in each group after backpack loading was minimal. However, the former showed a larger step width and a greater increase in contact area in the midfoot region, while the latter demonstrated a greater increase in peak pressure.

**Conclusions:**

Individuals with mild flatfoot demonstrated significantly different gait curve patterns (waveforms) compared to the controls. In the loaded condition, the CON and MLA groups may have adopted different strategies to maintain balance during gait. We suggest that although individuals with asymptomatic mild flatfoot are drafted as active-duty soldiers, they should be thoroughly investigated under loaded conditions, and orthoses may be helpful.

## Background

Flatfoot is a three-dimensional deformity characterized by a low-arch with hindfoot valgus, midfoot abduction, and forefoot supination [1, 2]. Patients with flatfoot deformity can complain of chronic foot pain and easy fatigue [3], and often have heel cord tightness [2] on physical examination.

Gait is closely related to foot posture. Previous studies have shown differences in the gait patterns of patients with flatfoot compared to normal controls [4, 5]. In addition, differences in the kinematics of the foot and ankle in proportion to the severity of the flatfoot deformity have been investigated [6]. Although the curve patterns for the kinematics of the severe flatfoot group (Meary angle over 20 degrees) were significantly distinct from those of the age- and sex-matched controls, the moderate flatfoot group (Meary angle between 10 and 20 degrees) demonstrated relatively closer trends to those in the control group [6]. Although Shin et al. cautiously suggested that the normal kinematic pattern may not collapse in cases of moderate flatfoot with a Meary angle of less than 20 degrees, they did not provide specific cut-off points [6].

Because the weight of the body is concentrated on the feet, which play a critical role in shock absorption and posture maintenance [7, 8], the effect of additional load on gait has been described in several prior studies [9–11]. According to Singh and Koh, an additional load of 20% of body weight led to a significant decrease in gait speed and cadence, and an increase in double support time [11]. Furthermore, previous research has revealed that low-arched individuals show greater arch deformation in response to load [12]. Son previously investigated the effect of backpack load on foot plantar pressure in individuals with flatfoot, and reported a significant increase in the contact area and pressure in the lateral and medial heel zones [13]. These methods could be useful strategies for balancing. However, pedobarography and gait analysis have not been simultaneously performed in previous studies, and the study participants were not limited to asymptomatic individuals with mild flatfoot.

In the authors’ country, all young adult males are required to undergo compulsory military service. Currently, military manpower administration in the authors’ country is deploying its personnel by setting the standards for the degree according to the disease state and disabilities. Individuals diagnosed with mild low-arched feet (talo-first metatarsal angle between 6 and 15 degrees in standing lateral radiographs), with or without symptoms, are drafted as active-duty soldiers [14]. However, individuals judged as having severe flatfoot (talo-first metatarsal angle greater than 15 degrees on a standing lateral radiograph) serve in the supplementary services. A previous study showed that anterior knee pain was more prevalent in individuals in the armed forces with flexible flat feet than in those with normal feet [15]. Kaufman et al. reported that flat feet are a risk factor for musculoskeletal overuse injury in military trainees [16], whereas Esterman and Pilotto demonstrated that individuals with flat feet had significantly poorer subjective physical health than those with normal feet [17].

A young person with mild flatfoot may experience an aggravation of symptoms or an increase in physical demand under the extra load of a backpack. Even if the lateral talo-first metatarsal angle does not coincide with the criteria for supplementary services at the moment of physical examination, an extra load, such as a backpack load of 20 kg, may exert a great impact on mild low-arched feet during gait. As such, the average weight of the backpack during military rucking, which is approximately 20 kg, was simulated in this study. The authors anticipated that gait and posture would be altered to maintain balance and distribute weight under these conditions.

To the best of our knowledge, no studies have yet investigated the effect of extra load on the intersegmental motions of the foot and ankle in asymptomatic individuals with mild flatfoot. This study therefore aimed to evaluate (1) the effects of a backpack load of 20 kg on gait and foot plantar pressures and (2) any possible differences based on the presence of mild flatfoot. We hypothesized that wearing a heavy backpack would alter the midfoot kinematics and plantar pressure pattern for shock absorption and posture maintenance, which may vary in individuals with mild flatfoot.

## Methods

### Study protocol

This was a level III prospective case-control study. All study participants provided informed consent, and the study protocol was approved by our institutional review board. Forty-eight asymptomatic male volunteers between the ages of 20 and 28 years, considered as candidates for active military duty, were locally recruited.

The inclusion criteria were as follows: (a) lateral talo-first metatarsal angle less than 15 degrees on standing foot radiograph: those with an angle between 8 and 15 degrees were classified in the mild low-arched (MLA) group, and those with an angle less than 6 degrees were classified as the control (CON) group [14, 18]; (b) absence of subjective symptoms such as foot and ankle pain or discomfort during gait; and (c) no observed radiographic features of progressive osteoarthritis. The exclusion criteria were as follows: (a) any neuromuscular disorders affecting the lower extremities, such as cerebral palsy; (b) spinal pathologies limiting activities of daily living; (c) other deformities, such as tarsal coalition and vertical talus; and (d) a history of surgery involving the lower extremities.

### Radiographic measurements

A standing lateral foot plain radiograph was obtained for each participant. Radiographic measurements were performed using a picture archiving and communication system (PACS) software (INFINITT PACS, INFINITT Healthcare Co., Seoul, Korea). The lateral talo-first metatarsal angle was used to evaluate flatfoot severity. Although the previous reference set the standard for angles between individuals with mild flatfoot and normal individuals at 6 degrees [14, 18], the authors set a value of 8 degrees to define mild flatfoot, to exclude errors and ambiguity in angle measurements. Following the measurement, participants were grouped into the MLA (*n* = 15) and CON (*n* = 15) groups. Of the 48 volunteers, 30 were finally enrolled in the study, and the remaining 18 were excluded as they fell in the gray zone between 6 and 8 degrees. Two graduate medical school students (PRB and JWL) and an orthopedic surgeon with 6 years of experience (MGK) measured the angles and calculated the interobserver reliability.

### Experimental procedures

All 30 participants completed pedobarography while 10 participants from each group agreed to complete foot gait analysis. The remaining 10 participants could not afford an appropriate schedule for a revisit to perform the gait analysis experiment; therefore, only the gait data of final 20 participants were used for analysis.

Gait and pedobarographic data were collected at the Human Motion Analysis Laboratory of the authors’ institution. We used the DuPont foot model to evaluate intersegmental foot motion [19, 20]. The placement of the markers, definition of the coordinate systems based on these markers, and method for calculating the joint rotation have all been previously described [21, 22].

The 15 markers were placed as follows: 2 markers on the knee (medial and lateral), 3 markers on the tibial shank (upper, front, and rear), 2 markers on the ankle (medial and lateral), 2 markers on the hindfoot segment (heel proximal and distal), 2 markers on the midfoot segment (navicular and cuboid), 3 markers on the forefoot segment (first metatarsal head, toe, and fifth metatarsal head), and 1 marker on the hallux [22]. This foot model consisted of the hindfoot, forefoot, first ray, fifth ray, and hallux. The relationship between segments was calculated in the sagittal, coronal, and axial planes from a ZXY Euler decomposition of the relative orientation of the anatomical coordinate systems, as previously described [23, 24]. The general orientation of the coordinate system is as follows: the X-axis, the vertical axis pointing down toward the plantar surface of the foot; the Y-axis, the anterior-posterior axis pointing forward; and the Z-axis, the medial-lateral axis pointing to the segment’s right [24].

The participants were asked to warm up for 5 minutes by walking at an easy pace. A single operator placed the reflective markers from the DuPont foot model [19]. Baseline static data were obtained in a calibration trial with feet positioned flat on the ground and parallel to each other on the coronal axis. The participants were subsequently asked to walk barefoot at their self-selected walking speed along a 9 m track. Gait data were collected using 12 cameras placed at a height of 2 m, with an optical motion capture system (Motion Analysis Co., Santa Rosa, CA, USA), at a sampling rate of 120 Hz. Eight cameras were set up at each octant position at 45° intervals. Four additional cameras were located on the back, front, and bilaterally. The resolution of the cameras was 1.3 megapixels with 500 frames/s. The distance between the cameras and the participants ranged from 3 to 7 m. The translational accuracy was 0.5 mm root mean square, and the angular resolution was 0.3°. The Cortex 1.3.0675 (Motion Analysis Co., Santa Rosa, CA, USA) was used for real-time motion capture, post-processing, and tracking of marker data.

**Fig. 1:**
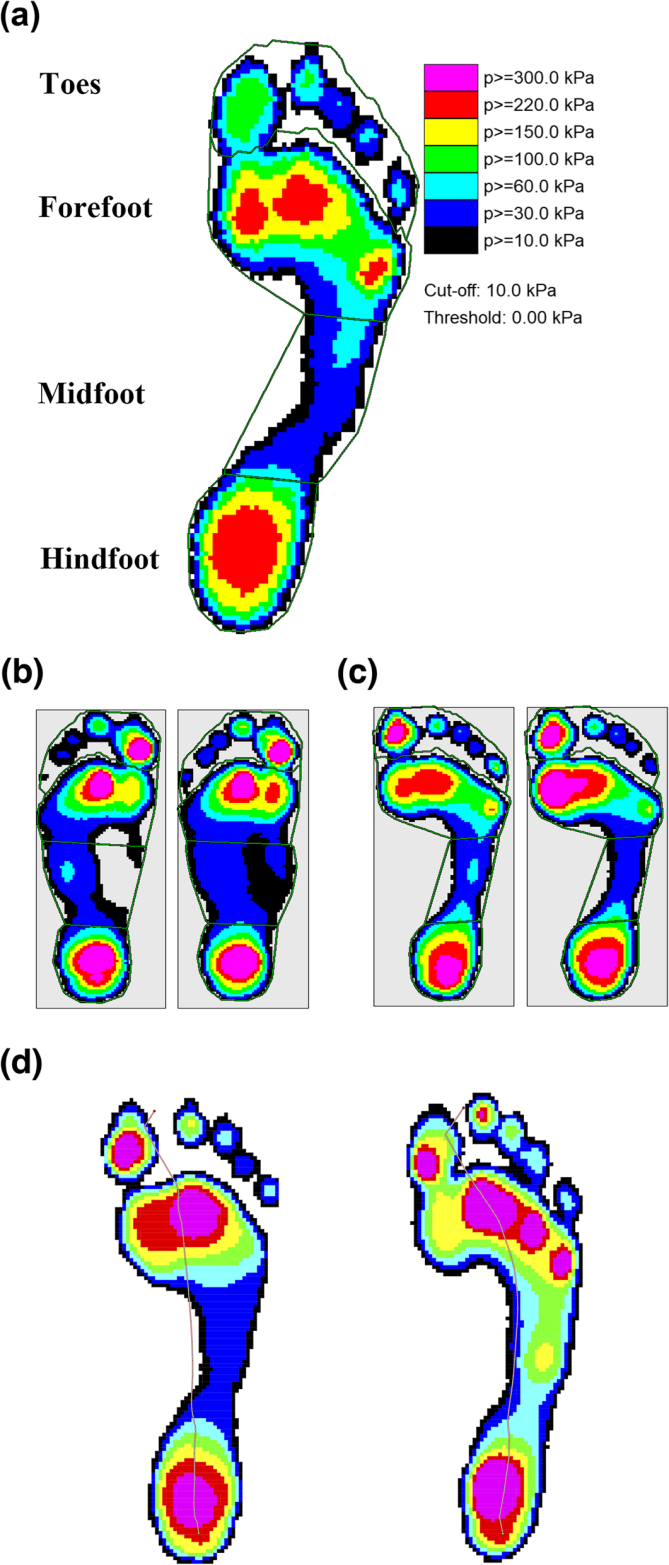
**a** Illustration of the four-mask standard division used in the analysis of plantar pressure. **b** Representative pedobarographic data of the mild low-arched group, at baseline (left) and with a 20-kg of backpack (right). **c** Representative pedobarographic data of the control group, at baseline (left) and with a 20-kg of backpack (right). **d** Center of pressure trajectories of the mild low-arched group (left) demonstrated greater medial deviation compared to the control group (right)

The emed® n50 pedobarography platform (novel GmbH, Germany) was used to record and evaluate the dynamic foot plantar pressure during gait. The plantar pressure data were acquired simultaneously with the gait trials, as the emed® n50 pedobarography platform was embedded along the track. The sensor area of this system was 475 × 320 mm^2^, with a resolution of 4 sensors/cm^2^, operating at 50 Hz. Participants were asked to walk barefoot at their usual speed on the platform. The emed® CL (novel GmbH, Germany) software was further used to analyze the plantar pressure distribution. Four regions (the hindfoot, midfoot, forefoot, and toes) were masked (Fig. 1A), and the contact area (cm^2^), maximum force (N), and peak pressure (kPa) were calculated for each region. The center of pressure excursion index was measured in both groups, as previously described [25].

Gait trials and pedobarography recordings were performed following the same procedures with the participants wearing a 20-kg backpack, which was equivalent to 28.4 ± 3.6% of the participants’ body weight. As the load distribution within the backpack may have an effect on gait [11], a homogenous metal disc of 20 kg was used for weighting to ensure equal load distribution.

### Gait data processing

Temporal and spatial gait parameters were obtained. To reduce the inter-individual variation due to body size, the speed (m/s), stride length (cm), and step width (cm) were divided by height (cm) and designated as n speed, n stride length, and n step width, respectively [26].

For the kinematic data analysis, three representative strides from five separate trials were selected based on the waveforms of the range of motion curve, excluding the maximum and minimum curves. Plantar pressure variables of the dominant foot matching the three representative strides were analyzed. To assess the intersegmental foot position (hindfoot relative to the tibia, forefoot to the hindfoot, and hallux to the forefoot) during the gait cycle, we divided the entire gait cycle into 100 points with a 1% interval and collected the intersegmental angles (ISAs) at each time point. The calculated parameters were as follows: (1) hindfoot relative to the tibia: dorsiflexion/plantarflexion (sagittal plane), pronation/supination (coronal plane), and internal/external rotation (transverse plane); (2) forefoot relative to the hindfoot: dorsiflexion/plantarflexion (sagittal plane), pronation/supination (coronal plane), and abduction/adduction (transverse plane); and (3) hallux relative to the forefoot: dorsiflexion/plantarflexion (sagittal plane) and valgus/varus (transverse plane). The data of the intersegmental positions of the first and fifth rays were recorded, but are not reported in the present study.

To compare the position of the foot and ankle segments between groups or states, the ISAs (position) in the middle of eight phases of gait (initial contact (IC) [0–2%], load response (LR) [6–8%], mid-stance (MS) [21–23%], terminal stance (TS) [40–42%], pre-swing (PSw) [55–57%], initial swing (ISw) [67–69%], mid-swing (MSw) [80–82%], and terminal-swing (TSw) [93–95%]) were measured, and the change in intersegmental angle (motion) between phases was calculated as previously described [24, 27, 28].

### Statistical analysis

The normality of the data was evaluated using the Shapiro–Wilk test. The Mann–Whitney U test was used to compare plantar pressure variables, spatiotemporal parameters, and intersegmental foot and ankle motion between the CON and MLA groups. The Wilcoxon signed-rank test was used to compare spatiotemporal parameters and intersegmental foot and ankle motion between baseline and extra-load states. The intraclass correlation coefficient was used to analyze the interobserver reliability of the radiographic measurements. IBM SPSS Statistics version 25 (Armonk, NY, USA) was used for all statistical analyses. Statistical significance was set at *P* <  0.05.

## Results

The participants’ demographic data are presented in Table 1. There were no significant differences between the two groups, except for the lateral talo-first metatarsal angle. The interobserver reliability of the measurement of this angle showed excellent agreement (intraclass correlation coefficient = 0.903 (*P* <  0.001).

**Table 1:** Participants’ demographic data

	Gait analysis			Pedobarography		
	CON (*n* = 10)	MLA (*n* = 10)	*P* value^a^	CON (*n* = 15)	MLA (*n* = 15)	*P* value^a^
Age, year	23.9 (1.4)	23.1 (1.9)	0.247	23.5 (1.6)	23.3 (2.0)	0.539
Height, cm	174.3 (4.3)	175.6 (4.2)	0.393	175.3 (4.3)	175.3 (4.6)	0.967
Weight, kg	70.6 (10.1)	72.6 (7.8)	0.912	69.7 (8.8)	72.9 (8.0)	0.567
Body mass index, kg/m^2^	23.2 (3.0)	23.6 (2.5)	0.739	22.7 (2.6)	23.7 (2.3)	0.461
Foot length, cm	25.3 (1.1)	26.1 (0.9)	0.075	25.5 (1.0)	25.9 (1.0)	0.325
Lateral talo-first metatarsal angle, degrees	0.17 (1.48)	12.5 (2.0)	< 0.001	−0.14 (1.39)	11.8 (2.4)	< 0.001

*Note:* Data are presented as mean value (standard deviation)

*Abbreviation*: *CON* Control group, *MLA* Mild low-arched group

^a^Mann–Whitney U test between CON and MLA group

In the hindfoot, the contact area, maximum force, and peak pressure tended to increase after loading in both groups; however, no significant changes were observed between the groups (Table 2). In the midfoot, the increase in contact area was significantly higher in the MLA group (*P* = 0.006) (Fig. 1B). In the forefoot, the increase in peak pressure was significantly higher in the CON group (*P* = 0.026) (Fig. 1C). Furthermore, the center of pressure trajectories in the MLA group demonstrated greater medial deviation and were less concave after loading (Fig. 1D). The center of pressure excursion index was 13.7 ± 9.0 in the MLA group and 18.6 ± 5.3 in the CON group (*P* = 0.039). The baseline maximum force in the toes was significantly higher in the MLA group (*P* = 0.016).

**Table 2:** Parameters measured by pedobarography

		Contact area, cm^2^		Maximum force, N		Peak pressure, kPa	
		Baseline	Difference^a^	Baseline	Difference^a^	Baseline	Difference^a^
Hindfoot	CON	35.00 (2.66)	0.58 (1.11)	447.50 (48.31)	94.11 (38.72)	318.33 (50.77)	77.67 (60.03)
	MLA	35.58 (2.72)	0.87 (0.68)	462.45 (64.41)	87.25 (36.02)	357.33 (105.16)	82.33 (65.22)
	*P* value^b^	0.838	0.325	0.436	0.389	0.250	0.624
Midfoot	CON	25.55 (7.49)	1.66 (2.66)	99.55 (42.85)	36.15 (46.97)	97.00 (29.02)	37.67 (36.64)
	MLA	29.77 (6.75)	5.43 (5.23)	115.14 (58.78)	55.83 (28.66)	108.67 (47.15)	34.33 (31.22)
	*P* value^b^	0.126	0.006	0.624	0.148	0.806	0.935
Forefoot	CON	52.86 (5.45)	1.73 (1.36)	577.08 (90.64)	169.04 (42.67)	444.67 (154.09)	241.00 (213.09)
	MLA	54.50 (5.93)	2.30 (2.36)	569.37 (67.60)	148.16 (35.66)	503.67 (150.35)	107.33 (100.60)
	*P* value^b^	0.595	0.461	0.389	0.187	0.106	0.026
Toes	CON	23.89 (4.23)	2.25 (2.02)	141.18 (57.53)	32.11 (35.10)	312.67 (143.43)	104.67 (133.01)
	MLA	25.53 (2.73)	1.70 (3.27)	184.23 (40.22)	29.58 (32.13)	420.00 (172.71)	74.00 (113.31)
	*P* value^b^	0.412	0.683	0.016	0.806	0.081	0.461

*Note:* Data are presented as the mean value (standard deviation)

*Abbreviation*: *CON* Control group, *MLA* Mild low-arched group

^a^The difference of each parameters after backpack loading minus baseline data

^b^Mann–Whitney U test between CON and MLA group

Regarding temporal gait parameters, we found no significant difference in the baseline (without backpack) temporal parameters between the two groups (Table 3). Cadence, gait speed, and stride length all decreased under the effect of extra load, regardless of flatfoot severity. The step time significantly increased after the extra load in both groups. Furthermore, the proportion of the stance phase significantly increased after backpack loading (CON: 62.0 to 63.8, *P* = 0.005; MLA: 62.4 to 63.9, *P* = 0.005) in both groups. In the heavy backpack loaded condition, the MLA group showed a significantly greater step width than the CON group (*P* = 0.019).

**Table 3:** Temporal gait parameters

	Study population							
	CON (*n* = 10)	CON + 20 kg (*n* = 10)	MLA (*n* = 10)	MLA + 20 kg (*n* = 10)	*P* value^a^	*P* value^b^	*P* value^c^	*P* value^d^
Cadence, step/min	113.8 (5.3)	110.7 (5.7)	111.4 (5.2)	109.6 (6.0)	0.022	0.028	0.315	0.796
Speed, m/s	1.28 (0.10)	1.20 (0.08)	1.22 (0.09)	1.16 (0.08)	0.007	0.005	0.579	0.579
n Speed^e^	0.74 (0.06)	0.69 (0.05)	0.70 (0.05)	0.67 (0.05)	0.007	0.009	0.353	0.393
Stride length, cm	134.8 (6.9)	130.4 (4.7)	131.4 (11.2)	128.0 (8.0)	0.028	0.013	0.280	0.436
n Stride length^e^	77.4 (4.1)	74.8 (2.7)	74.9 (6.0)	73.1 (4.5)	0.028	0.047	0.280	0.393
Step width, cm	12.4 (1.9)	12.4 (2.2)	13.5 (1.8)	14.4 (2.1)	0.878	0.285	0.143	0.019
n Step width^e^	7.1 (1.0)	7.1 (1.1)	7.7 (1.1)	8.2 (1.1)	0.878	0.285	0.218	0.011
Step time, s	0.53 (0.02)	0.54 (0.03)	0.54 (0.03)	0.55 (0.03)	0.022	0.028	0.315	0.796
Proportion of stance phase, %	62.0 (1.5)	63.8 (0.9)	62.4 (1.1)	63.9 (0.9)	0.005	0.005	0.739	1.000

*Note:* Data are presented as the mean value (standard deviation)

*Abbreviation*: *CON* Control group, *MLA* Mild low-arched group

^a^Wilcoxon signed rank test between the baseline and 20 kg backpack load state in CON group

^b^Wilcoxon signed rank test between the baseline and 20 kg backpack load state in MLA group

^c^Mann–Whitney U test between the CON and MLA group in baseline state

^d^Mann–Whitney U test between the CON and MLA group in 20 kg backpack load state

^e^Normalized with the subject’s height (speed, stride length, and width divided by subject’s height)

**Fig. 2:**
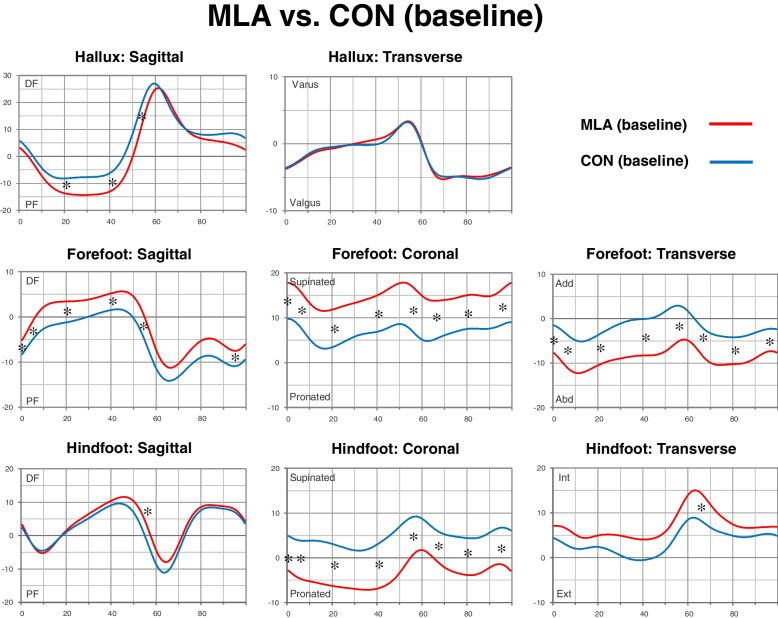
Average kinematics of the hallux relative to the forefoot, forefoot relative to the hindfoot, and hindfoot relative to the tibia during the entire gait cycle. Comparison of the mild low-arched (MLA) group with control (CON) group at baseline state is shown. The horizontal axis represents the gait cycle and the vertical axis represents the range of motion. Asterisks denote a significant change between the two groups. Abd, abduction; Add, adduction; DF, dorsiflexion; Ext, external rotation; Int, internal rotation; PF, plantar flexion

**Fig. 3:**
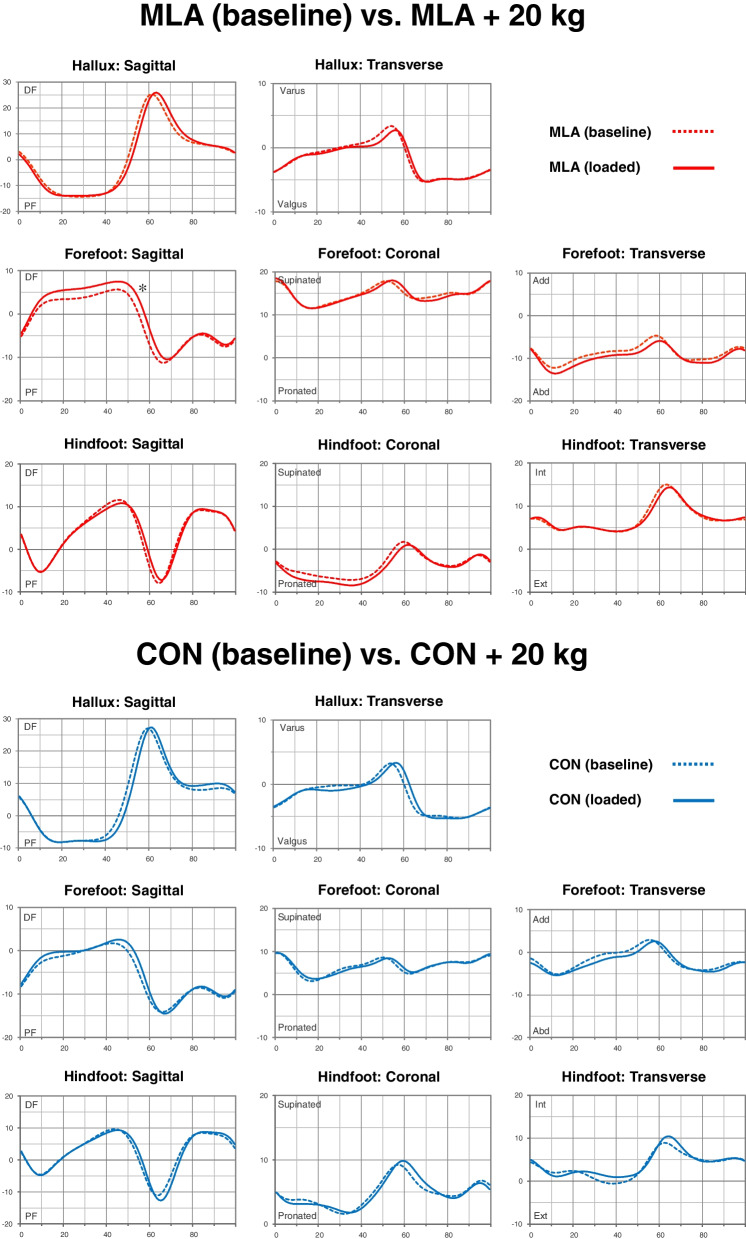
Average kinematics of the hallux relative to the forefoot, forefoot relative to the hindfoot, and hindfoot relative to the tibia during the whole gait cycle. Comparison of the mild low-arched (MLA) group at baseline (red dotted line) and in backpack loaded (red solid line) state is shown. Comparison of the control (CON) group at baseline (blue dotted line) and in backpack loaded (blue solid line) state is shown. The horizontal axis represents the gait cycle and the vertical axis represents the range of motion. An asterisk denotes a significant change between the two states. Abd, abduction; Add, adduction; DF, dorsiflexion; Ext, external rotation; Int, internal rotation; PF, plantar flexion

The ISAs of the foot segment relative to the proximal segment in each phase of the entire gait cycle are presented in Figs. 2 and 3.

We compared the baseline (without backpack) foot kinematics of the asymptomatic mild low-arched group with those of the age- and sex-matched control groups (Fig. 2). In the hallux kinematics relative to the forefoot, the MLA group showed significant plantar flexion (MS, *P* <  0.001; TS, *P* <  0.001; and PSw, *P* = 0.035) (Table 4). Regarding forefoot kinematics relative to the hindfoot, the MLA group showed significant supination throughout the gait cycle. Sagittal motion further revealed that the MLA group had a greater maximal dorsiflexion than the CON group. Forefoot abduction was further observed throughout the gait cycle in the MLA group. In hindfoot kinematics relative to the tibia, the coronal position of the MLA group showed more pronation throughout the gait cycle than that of the CON group. There was also a tendency for dorsiflexed position in the MLA group; however, this was significant only in the PSw phase (*P* = 0.023). Furthermore, there was a tendency for internal rotated position in the MLA group; however, a significant change was found only in the ISw phase (*P* = 0.009).

**Table 4:** Comparison of the average kinematics during gait cycle between the mild low-arched (MLA) group with control (CON) group at baseline state

		Gait cycle phases							
		IC	LR	MS	TS	PSw	ISw	MSw	TSw
Hallux	Sagittal	0.393	0.315	< 0.001	< 0.001	0.035	0.971	0.684	0.218
	Transverse	0.739	0.912	0.853	0.739	0.971	0.280	0.796	0.796
Forefoot	Sagittal	0.011	< 0.001	0.019	0.001	0.019	0.190	0.052	0.015
	Coronal	0.015	0.011	0.035	0.005	< 0.001	0.015	0.009	0.015
	Transverse	0.009	0.007	0.011	0.001	0.004	0.011	0.009	0.009
Hindfoot	Sagittal	0.481	0.529	0.739	0.315	0.023	0.393	0.529	0.436
	Coronal	0.029	0.009	0.019	0.001	0.004	0.009	0.003	0.002
	Transverse	0.123	0.075	0.280	0.063	0.075	0.009	0.105	0.579

*Note:* Data are presented as *P* values (Mann–Whitney U test between the CON and MLA group)

*Abbreviations*: *IC* initial contact, *ISw* initial swing, *LR* load response, *MS* mid-stance, *MSw* mid-swing, *PSw* pre-swing, *TS* terminal stance, *TSw* terminal-swing

When we compared the baseline foot kinematics of the asymptomatic mild low-arched group with that of the backpack-loaded state, we found no significant differences in hallux kinematics relative to the forefoot and hindfoot kinematics relative to the tibia (Fig. 3). In forefoot kinematics relative to the hindfoot, sagittal motion showed more dorsiflexion only in the PSw phase (*P* = 0.009). The effect of extra load on the gait pattern was not significant in any segment in the CON group.

## Discussion

In this study, we performed gait analysis and pedobarography on asymptomatic young male participants and revealed that (1) the change in intersegmental foot and ankle motion following backpack loading was minimal and (2) intersegmental foot and ankle motion differed based on the presence of mild flatfoot. Furthermore, the MLA group demonstrated a significantly higher contact area of the midfoot and less increased forefoot peak pressure compared to the CON group when carrying a heavy backpack.

Shock absorption and posture maintenance play essential roles in gait [7, 8]. Therefore, it is crucial to understand the effects of heavy loads on gait. The effect of extra load on gait patterns has been previously reported [9–12]. According to Singh and Koh, an additional load of 20% of body weight led to a significant decrease in gait speed and cadence, concurrent with an increase in double support time [11]. Our data similarly demonstrated that regardless of the presence of mild flatfoot, cadence, gait speed, and stride length decreased, and the step time and proportion of the stance phase increased significantly under extra load. This could be a strategy to dispense weight. In other words, under the backpack load, the participants made a greater effort to maintain balance and take more cautious steps. In addition, an increased step width was observed in the MLA group, which may have contributed to making balance.

In our study, despite alterations in temporospatial parameters following the loaded condition, the effect of a heavy backpack load on the intersegmental foot and ankle motions of each group was minimal. This may be explained by the short duration of loading or the threshold for arch deformation, depending on the weight. A load greater than 20 kg with a longer duration may show different results. However, we chose a load of 20 kg in the backpack to simulate military rucking and to ensure the safety of the participants. Further studies are required to evaluate the dose-response relationship of loading in gait. Additionally, the limitation of the current multi-segment foot model used in this study may have contributed to the less meaningful effect of backpack load. Although earlier studies have reported high repeatability [21, 22] and differences were observed between the two populations at baseline, the marker-based approach was not capable of capturing the subtle yet significant difference in foot and ankle kinematics due to load. More refined marker sets combined with other techniques, such as biplanar fluoroscopy, weight-bearing computed tomography, or dynamic magnetic resonance imaging, may aid in this regard.

Flatfoot can be easily diagnosed radiographically by an observation of the collapse of the medial longitudinal arch. Severity is often evaluated using the lateral talo-first metatarsal angle on standing radiographs [29, 30]. According to a gait analysis of symptomatic patients with flatfoot [6], the severe flatfoot group showed a significantly supinated and abducted position of the forefoot and significantly pronated and adducted position of the hindfoot throughout the gait cycle. However, Shin et al. suggested that the normal kinematic pattern might not collapse in moderate flatfoot with a Meary angle of less than 20 degrees [6]. Our data are distinct from these results in that the MLA group showed significantly different intersegmental kinematics, especially in the forefoot relative to the hindfoot, compared with the CON group.

When considering both load and the presence of flatfoot, according to Zifchock et al., individuals with a low arch showed greater arch deformation in response to a load [12]. In contrast, Powell et al. insisted that high- and low-arched athletes showed no differences in deformation of the medial arch when vertical loading was applied [10]. In the current study, the contact area, maximum force, and peak pressure in the hindfoot tended to increase after loading in both groups; however, no significant differences between the groups were observed. These findings agree with those of a previous study which reported a significant increase in the contact area and pressure of the lateral and medial heel zones under backpack load [13]. In the midfoot, although the baseline contact area was similar between the two groups, the increase was significantly higher in the MLA group. Furthermore, the center of pressure trajectories in the MLA group were found to be less concave, indicating a greater medial deviation. Meanwhile, in the forefoot, the increase in peak pressure was significantly higher in the CON group. Together, these results show that different strategies were utilized by each group to dispense weight during gait. A previous study reported that loading of 15 to 20% of bodyweight led to a significantly higher trunk forward lean [11]. We therefore presume that participants in the MLA group would lean forward and increase their forefoot dorsiflexion and step width to make the center of mass of the backpack together with the body well within the boundary of support. The participants in the CON group may also have leaned forward, leading to an increase in peak pressure in the forefoot; however, future studies are required to validate the shifting of the trunk, as we did not use markers on the trunk in this study.

The main reason the authors conducted this study is that although individuals with mild-low-arched feet were asymptomatic at the baseline state, we empirically anticipated that they may later experience different levels of increase in physical demand under the extra load of a backpack, as patients with flatfoot often complain of chronic foot pain and easy fatigue [3]. Furthermore, we hypothesized that under a heavy load, different arch configuration attributes to demonstrate different strategies to maintain balance and distribute weight, which may be reflected in the gait curve and foot plantar pressure pattern. Contrary to our expectations, it was not possible to directly infer that asymptomatic patients with mild flatfoot are prone to injuries and diseases, such as stress fractures. However, under loaded conditions during foot contact, from heel strike to foot flat and toe-off phases, a greater increase in midfoot contact area and intersegmental motions of increased dorsiflexion of the forefoot relative to the hindfoot possibly suggest greater arch deformation in the MLA group. Therefore, the results of our study may serve as a rationale for providing orthosis, such as medial arch supporting insoles, to help MLA individuals who need to wear a heavy backpack. Previous studies also support our hypothesis that arch-supporting insoles aid in preventing further collapse of the medial longitudinal arch, redistributing pressure, and inducing load transfer from the heel to the midfoot region [31, 32].

This study highlights that the effect of extra load in asymptomatic individuals with mild flatfoot was evaluated by intersegmental motions using a multi-segment foot model as well as dynamic pedobarographs. However, this study has several limitations. First, although this study aimed to simulate military rucking, gait analysis using 15 skin markers and pedobarographic measurements were conducted barefoot. However, military personnel wear sturdy boots during practical training, which will undoubtedly affect foot and ankle function. Therefore, caution should be exercised when interpreting the study results in military practice. Second, we included a relatively small number of young male volunteers, only a subset of whom completed the gait analysis. Although a previous study demonstrated that there are sex differences in gait patterns [27], this study primarily focused on male participants who are candidates for military personnel. Third, a radiographic assessment of arch deformation after backpack loading was not performed. Future research should compare radiographic results with the functional modalities used in this study. Fourth, load distribution inside the backpack was not considered, although a previous study reported the effect of backpack load position on gait parameters [11]. Our study primarily focused on the possible differences based on the presence of mild flatfoot. In addition, all participants wore the same backpack, to control variability. Lastly, the flexibility or rigidity of the foot was not assessed as it is important in response to a load. However, as this study included asymptomatic individuals with mild flatfoot as participants, the authors believe that flexibility or rigidity would not have significantly affected the results.

## Conclusions

In the loaded condition, although temporal parameters such as cadence, gait speed, stride length, and proportion of the stance phase changed significantly, the change in the intersegmental foot and ankle motion was minimal. The gait pattern differed based on the presence of mild flatfoot. In particular, the MLA group demonstrated several strategies to maintain balance when wearing a heavy backpack. They showed a larger step width, a greater increase in contact area of the midfoot region which resulted in less increased forefoot peak pressure, and increased dorsiflexion of the forefoot relative to the hindfoot segment, potentially demonstrating greater arch deformation than that of the CON group. Therefore, the authors suggest that although individuals with asymptomatic mild flatfoot are drafted as active-duty soldiers, they should be thoroughly investigated under loaded conditions, and orthoses such as medial arch supporting insoles may be helpful.

## Data Availability

The datasets used and analyzed during the current study are available from the corresponding author on reasonable request.

## Abbreviations

CON: control
IC: initial contact
ISA: intersegmental angles
ISw: initial swing
LR: load response
MS: mid-stance
MSw: mid-swing
MLA: mild low-arched
PSw: pre-swing
TS: terminal stance
TSw: terminal-swing
